# Genetic fecal source identification in urban streams impacted by municipal separate storm sewer system discharges

**DOI:** 10.1371/journal.pone.0278548

**Published:** 2023-01-26

**Authors:** Adam Diedrich, Mano Sivaganesan, Jessica R. Willis, Amirreza Sharifi, Orin C. Shanks

**Affiliations:** 1 U.S. Environmental Protection Agency, Office of Research and Development, Cincinnati, OH, United States of America; 2 Department of Energy and Environment, Government of the District of Columbia, Washington, DC, United States of America; Cranfield University, UNITED KINGDOM

## Abstract

Municipal stormwater systems are designed to collect, transport, and discharge precipitation from a defined catchment area into local surface waters. However, these discharges may contain unsafe levels of fecal waste. Paired measurements of *Escherichia coli*, precipitation, three land use metrics determined by geographic information system (GIS) mapping, and host-associated genetic markers indicative of human (HF183/BacR287 and HumM2), ruminant (Rum2Bac), dog (DG3), and avian (GFD) fecal sources were assessed in 231 urban stream samples impacted by two or more municipal stormwater outfalls. Receiving water samples were collected twice per month (*n* = 24) and after rain events (*n* = 9) from seven headwaters of the Anacostia River in the District of Columbia (United States) exhibiting a gradient of impervious surface, residential, and park surface areas. Almost 50% of stream samples (*n* = 103) were impaired, exceeding the local *E*. *coli* single sample maximum assessment level (410 MPN/100 ml). Fecal scores (average log_10_ copies per 100 ml) were determined to prioritize sites by pollution source and to evaluate potential links with land use, rainfall, and *E*. *coli* levels using a recently developed censored data analysis approach. Dog, ruminant, and avian fecal scores were almost always significantly increased after rain or when *E*. *coli* levels exceeded the local benchmark. Human fecal pollution trends showed the greatest variability with detections ranging from 9.1% to 96.7% across sites. Avian fecal scores exhibited the closest connection to land use, significantly increasing in catchments with larger residential areas after rain events (p = 0.038; *R*^2^ = 0.62). Overall, results demonstrate that combining genetic fecal source identification methods with GIS mapping complements routine *E*. *coli* monitoring to improve management of urban streams impacted by stormwater outfalls.

## Introduction

An estimated 55% of the world population lives in urban areas [1]. Urban areas are built environments composed of elements such as homes, buildings, green spaces, streets, and sidewalks. These areas typically include elaborately engineered drainage networks collecting and directing stormwater runoff into local streams, often requiring minimal rain accumulation. Discharge from these municipal separate stormwater systems may contain unsafe levels of fecal pollutants originating from various sources ranging from illicit sewer connections to wildlife waste. Numerous studies report that rivers and streams that flow through urban areas are often impacted by fecal pollution to a greater extent compared to respective upstream, less populated locations [2, 3]. In addition, there is a large body of research identifying stormwater runoff as a vector of human pathogens [4, 5] and as a key contributor of fecal pollution [6–8] in urban waters. Urban stream microbial water quality is typically monitored with general fecal indicator bacteria, such as *Escherichia coli*, which provides information on the estimated total level of fecal pollution in a sample as well as indication of potential public health risk [9]. However, these measurements do not provide information on specific fecal pollutant sources. This limitation is often more pronounced in urban areas due to dense human, pet, and wildlife populations living near each other in areas with a high proportion of impervious surfaces (i.e., streets, sidewalks, roof tops), facilitating the rapid movement of stormwater and microbial contaminants from the city landscape into nearby natural receiving waters.

In response, the research community has developed numerous quantitative fecal source identification methodologies to compliment routine general fecal indicator bacteria monitoring offering the option to characterize fecal source pollution trends from key animal groups and potential public health risk results in parallel [10–14]. Many of these methods have been successfully used to characterize fecal pollution sources in natural waters impacted by rainfall [7, 15–17], but mostly focusing on human and/or dog waste contamination. However, less is known about the potential influence of stormwater outfall likely containing fecal matter from urban wildlife. Many cities are home to ducks, geese, and other waterfowl representing potential sources of general fecal indicators [18]. The GFD avian-associated fecal source identification method is reported to detect a broad range of waterfowl and other bird species [19, 20]. In addition, ruminant-associated methods can be used to track wildlife when there is confidence that sampling sites of interest are not impacted by agricultural ruminants (i.e., cattle, goats, sheep), such as urban catchments containing headwaters where animal feeding operations are typically not found. The Rum2Bac method is reported to be highly specific [21–23] and shown to detect at least 14 ruminant sources including wildlife species such as white-tail deer, mule deer, among others [23]. Together these methods provide an opportunity to assess human, dog, and wildlife impacts on urban streams impacted by stormwater outfall discharges.

This study evaluates paired measurements of host-associated genetic markers, *E*. *coli*, and precipitation from seven headwater urban streams situated at the southern portion of the Anacostia River Watershed (District of Columbia, U.S.A.) over a 13-month period. Unlike most highly urbanized areas, the District of Columbia is composed of approximately 27.9 km^2^ of park land, further complicating management due to the presence of indigenous avian and ruminant wildlife populations. In addition, each stream is impacted by two or more municipal stormwater outfalls and stream catchments that represent a gradient of land use based on geographic information system (GIS) measurements of impervious surface, residential, and park surface areas. A panel of five host-associated genetic markers designed to characterize human (HF183/BacR287 and HumM2), dog (DG3), avian (GFD), and ruminant (Rum2Bac) fecal sources are employed to characterize fecal pollution trends. Potential links between fecal source identification measurements, *E*. *coli*, precipitation, and land use are explored with a recently developed Bayesian qPCR censored data analysis approach [24] that has been successfully used to rank recreational water sites based on human fecal pollution levels [24, 25] and identify links between fecal indicator bacteria levels and host-associated genetic marker measurements [17, 26]. Findings indicate that human waste and urban wildlife both contribute to water quality in the District of Columbia and demonstrates that combining fecal source identification with GIS mapping and censored data analysis complements routine *E*. *coli* monitoring to improve water quality management in urban areas.

## Materials and methods

### Catchment and sampling site descriptions

**Fig 1:**
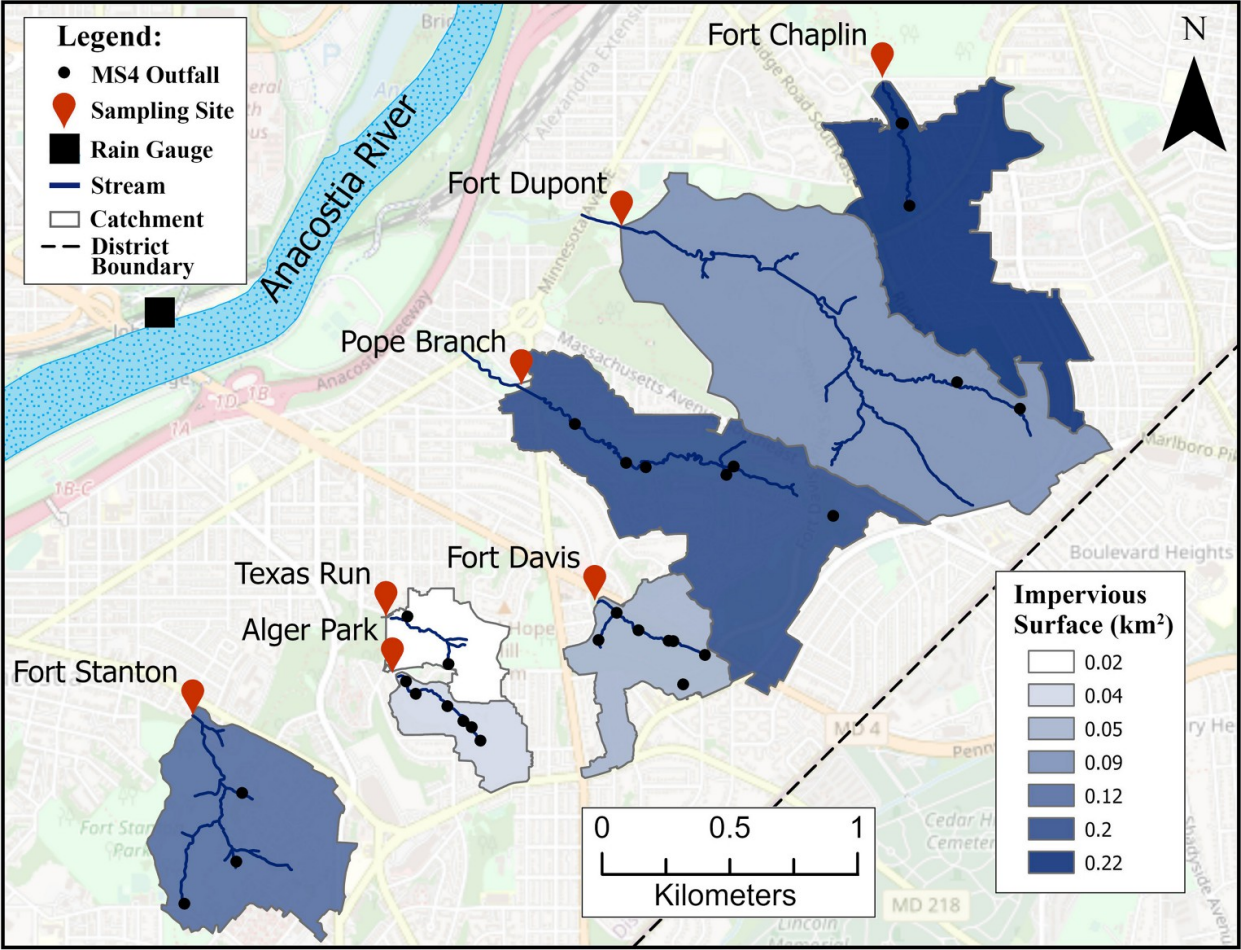
Geographic information system (GIS) map showing size and location of seven catchments included in study. Sampling locations are denoted with a red symbol. The rain gauge is indicated by a black shaded square. Catchments are colored based on total impervious surface area (km^2^). See legend for stream and catchment boundary information. The underlaying street map is from OpenStreetMap (Data available under the Open Database License: https://www.openstreetmap.org/copyright).

The Anacostia River Watershed is a 456 km^2^ area situated in the District of Columbia and Maryland (United States). Seven catchments from the southern portion of the watershed located in the District of Columbia city limits were selected for water quality testing including Fort Chaplin, Fort Dupont, Pope Branch, Fort Davis, Texas Run, Alger Park, and Fort Stanton (**Fig 1**). Catchments ranged in size from 0.13 (Alger Park) to 1.23 (Fort Dupont) km^2^ with a gradient of land use practices ranging from park land to residential. Catchment boundaries were defined with the Spatial Analysis Hydrology tool using stream and elevation data from the National Hydrology Dataset (http://datagateway.nrcs.usda.gov) combined with local stormwater transit pipe data (Department of Energy and Environment, Washington, DC). There are no septic systems or combined sewer overflow discharge points in catchments, all human waste is managed via centralized sewage systems. Each catchment contains two (Fort Dupont and Texas Run) to seven (Fort Davis) stormwater discharge outlets (Fig 1). Sampling sites were strategically located at the catchment outlet point where a respective headwater stream intersects the catchment boundary (Fig 1).

### Precipitation and land use characterization

The delivery and magnitude of fecal pollutants into receiving streams can be strongly influenced by local rainfall, especially in catchments with multiple stormwater drainage points [27, 28]. To measure precipitation, a reference gauge (WH24B Wireless Weather Station, Fine Offset Electronics, Ltd) was selected based on proximity to catchments and completeness of hourly measurements over the study period (**Fig 1**). Due to the nature and impact of precipitation events on urban catchments, cumulative precipitation 12h prior to a sampling event (mm) was used for all statistical analyses. ArcGIS ArcMap (version 10.3; ESRI, Redlands, CA) was employed to define catchment land use metrics including areas of impervious surface, park, and residential (km^2^) using the local management authority shape files (Department of Energy and Environment, Washington, DC).

### Water sampling

A total of 231 water samples were collected across 33 sampling events over 57-weeks from November 14, 2019 to December 15, 2020. Routine sampling events occurred twice per month. To increase the number of samples impacted by recent storms, seven additional sampling events were conducted immediately after cumulative rainfall from a single storm exceeded 2.54 mm following a 72 h dry period based on United States Environmental Protection Agency recommended storm event sampling guidance [29]. For each sampling event (total count = 33), water was collected from all sites on the same day within a short period of time (< 6 h). Samples were collected in sterile 1L containers from surface water and immediately stored on ice during transport to the laboratory. In addition, a field blank sample with molecular grade water substituted for receiving water was included for each sampling event.

### *E*. *coli* measurements

*E*. *coli* enumeration was performed within eight hours of sample collection with Colilert IDEXX defined substrate technologies (IDEXX Laboratories, Inc. Westbrook, ME). To assess water quality trends, a single sample maximum concentration of 410 most probable number (MPN) per 100 ml was used based on District of Columbia Department of Energy and Environment practices [30].

### Reference DNA preparations

Reference DNA sources consisted of two plasmid constructs and salmon sperm DNA. Plasmid constructs included the National Institute of Standards and Technology Standard Reference Material^®^ 2917 (SRM 2917; Rockville, MD) for qPCR calibration model generation [31, 32] and an internal amplification control (IAC; Integrated DNA Technologies, Coralville, IA). SRM 2917 is a commercially available, ready to use, linearized plasmid consisting of five dilution preparations as follows: Level 1 (10.3 copies/2 μL), Level 2 (1.11·10^2^ copies/2 μL), Level 3 (1.06·10^3^ copies/2 μL), Level 4 (1.06·10^4^ copies/2 μL), and Level 5 (1.04·10^5^ copies/2 μL). The IAC plasmid was linearized by ScaI-HF restriction digest (New England BioLabs, Beverly, MA), purified via QIAquick PCR Purification Kit (Qiagen, Valencia, CA), quantified with a Qubit dsDNA HS assay kit on a Qubit 3 Fluorometer (Thermo Fisher Scientific, Grand Island, NY), and diluted in 10 mM Tris 0.5 mM EDTA (pH 9.0) to generate 10^2^ copies/2μL. A salmon sperm DNA working stock containing 10 μg/ml was prepared by diluting a commercially available 10 mg/ml solution (Sigma-Aldrich, St. Louis, MO). All reference DNA material preparations were stored in low-adhesion microtubes either at 4°C (SRM 2917) or -20°C (IAC).

### Water filtration and DNA extraction

A total of 231 water samples were filtered for DNA extraction. For each sample, 100 ml was filtered through a 0.45 μm polycarbonate filter (Fisher Scientific, Pittsburg, PA). Filters were placed in a sterile 2 ml screw cap tube containing silica bead mill matrix (GeneRite, North Brunswick, NJ) and stored at -80°C (< 18 months) until DNA extraction. DNA extraction was performed with the DNA-EZ RW02 kit (GeneRite) as previously described [25]. Briefly, 600 μL of 0.02 μg/ml salmon sperm DNA (Sigma-Aldrich) was added to each bead mill tube followed by homogenization with a MP FastPrep-24 (MP Biomedicals, LLC Solon, OH) at 6.0 m/s for 30 s. Three method extraction blanks (MEB), with molecular grade water substituted for receiving water sample, were performed for each sample processing batch (12 samples/batch). DNA was eluted with 100 μL elution buffer into GeneMate Slick low-adhesion microcentrifuge tubes (ISC BioExpress, Kaysville, UT) and stored at 4°C prior to qPCR amplification (< 24 h).

### qPCR amplification

Fecal source identification employed five host-associated qPCR assays targeting four animal groups; namely human (HF183/BacR287 and HumM2), ruminant (Rum2Bac), dog (DG3), and avian (GFD) fecal sources [19, 21, 33–35]. The Sketa22 qPCR assay was also included as a sample processing control (SPC) [36]. All reactions contained 1X TaqMan Environmental Master Mix (version 2.0; Thermo-Fisher Scientific), 0.2 mg/ml bovine serum albumin (Sigma-Aldrich), 1 μM each primer, and 80 nM 6-carboxyfluoroscein (FAM)-labeled probe, and 80 nM VIC-labeled probe (HF183/BacR287 and HumM2 assays only). All reactions contained either 2 μL of DNA water sample extract or SRM 2917 in a total reaction volume of 25 μL. HF183/BacR287 and HumM2 multiplex reactions also contained 10^2^ copies of IAC template. Triplicate reactions were performed for SRM 2917 containing reactions and all Sketa22 testing while all fecal source identification experiments included six replicate reactions. Amplifications were conducted on a QuantStudio 6 Flex Real-Time PCR System (Thermo Fisher Scientific) with the following thermal cycle profile: 10 min at 95°C followed by 40 cycles of 15 s at 95°C and 1 min at 60°C. The threshold was manually set to either 0.03 (HF183/BacR287, DG3, Rum2Bac, and Sketa22) or 0.08 (HumM2 and GFD). Quantification cycle (C_q_) values were exported for further analysis. To monitor for potential extraneous DNA contamination during qPCR amplification, six no-template controls (NTC) with molecular grade water substituted for template DNA were performed with each instrument run.

### qPCR data acceptance metrics

The following data acceptance scheme was used to ensure high quality molecular data in this study. All fecal source identification qPCR assays were subject to calibration model acceptance criteria for linearity (*R*^2^ ≥ 0.980) and amplification efficiency (0.90 to 1.10, *E* = 10^(-1/slope)^ -1) [37, 38]. HF183/BacR287 and HumM2 multiplex IAC protocols were used to monitor for amplification inhibition in each water sample [34, 35]. Any DNA extract with evidence of amplification inhibition was discarded from the study. Instrument run-specific IAC proficiency testing (HF183/BacR287 and HumM2 NTC VIC C_q_ standard deviations ≤ 1.16 or 1.05, respectively) were also conducted to confirm reliable application of amplification inhibition testing from one instrument run to another [39]. A SPC protocol was implemented to monitor for suitable DNA recovery for each receiving water sample and to ensure consistent DNA recovery from one sample batch to another. Samples with unacceptable DNA recovery were excluded from the study based on batch-specific criteria derived from repeated MEB spike recovery measurements. SPC proficiency was determined for each sample batch requiring a standard deviation in Sketa22 qPCR MEB repeated measures of ≤ 0.62 C_q_ [39].

### Calculations and statistics

#### qPCR calibration model and performance

‘Mixed’ calibration models were generated for each fecal source identification qPCR assay and instrument run combination using a Bayesian Markov Chain Monte Carlo approach [40]. The lower limit of quantification (LLOQ) was defined as the instrument run specific 95% credible interval upper-bound from repeated measures (*n* = 18) of SRM 2917 dilution level 1. Outliers were defined as the absolute value of a studentized residual > 3.

#### Fecal score model

The fecal score approach [24], a qPCR censored-data method was used to rank sites by fecal pollution source and investigate potential linkages between host-associated genetic marker measurements, rainfall, and *E*. *coli* levels with the following modifications. A fecal score is a host-associated genetic marker weighted average concentration [log_10_ copies per 100 ml with 95% Bayesian credible interval (BCI)] calculated from a defined set of sample results including non-detections (C_q_ = 40, ND), detections below the LLOQ (below detection, BD), and measurements within the range of quantification (ROQ). For each set of water samples, C_q_ measurements were classified into three groups: ROQ group, BD group, or ND group. For the BD group, a Poisson distribution with a mean parameter λ_1_ (1 ≤ λ_1_ ≤ UB) was assumed for the number of genetic marker copies Z_1_, where the upper-bound (UB) was the number of copies corresponding to the respective qPCR assay LLOQ. For the ND group, the number of copies was assumed to be less than one and governed by a Poisson distribution with mean λ_2_ (λ_2_ < 1). For the ROQ group, a ‘mixed’ calibration model [40] was used to estimate the number of copies Z_3_ (log_10_ base). For a given sample Y from the i^th^ instrument run, posterior distributions of Z_1_, Z_2_ and Z_3_ were used to estimate the concentration X (log_10_ base). The Bayesian models used for Z_1_, Z_2_ and Z_3_ are given below:

For C_q_ measurements in the BD group,

Z_1_ ~ Poisson (λ_1_) · I (1,UB_i_)

UB_i_ = $10^{({LLOQ}_{i}-\alpha_{i})/\beta }$

log_10_ (λ_1_) ~ N (0, 10^3^) · I (0,).

For C_q_ measurements in the ND group,

Z_2_ ~ Poisson (λ_2_) · I (0, 1)

log_10_ (λ_2_) ~ N (0, 10^3^) · I (-2, 0).

For C_q_ measurements in the ROQ group,

log_10_ (Z_3_) = $\left\{ \begin{array}{c} {(C}_{q1}-\alpha_{i})/\bar{\beta }\;if\;r_{3}=1\\ {(C}_{qr}-\alpha_{i})/\bar{\beta }\;if\;r_{3}>1 \end{array}\right.$

*C*_*qr*_~ N (${\bar{C}}_{q}$, s^2^).

A weighted-average fecal score is then given in Eq 1,

$$Log_{10}(X_{j})=[r_{1}\cdot log_{10}(Z_{1})+r_{2}\;log_{10}(Z_{2})+r_{3}\cdot log_{10}(Z_{3})]/(r_{1}+r_{2}+r_{3})$$ (Eq 1)

where r_1,_ r_2_ and r_3_ are the number of C_q_ replicate measurements in each BD, ND and ROQ group, respectively, α_i_ is the intercept parameter for i^th^ instrument run and $\bar{\beta }$ is the master slope parameter of the mixed model calibration curve, ${\bar{C}}_{q}$ is the mean and s is the standard deviation of ${\bar{C}}_{q}$ derived from all measurements in the ROQ group. In addition, I(a,b) restricts the Poisson and Normal distributions to a range from a to b and I(0,) restricts these distributions to positive values. For given samples Y_1_, Y_2_,…,Y_m_, the posterior distribution of Fecal Score can be used to estimate the mean and the corresponding 95% BCI, where

$$Fecal\;Score=\sum_{1}^{m}\;{log}_{10}(X_{j})/m$$ (Eq 2)

For each water sample, six replicates were tested to ensure an adequate number of observations for suitable fecal score estimation [24]. A sample group was eligible for fecal score determination if it had 1) at least three samples (3 samples x 6 replicates = 18 data points), and 2) at least one replicate per sample must be from the BD or ROQ group (data sets consisting of all ND were not eligible).

To rank sites, fecal scores were calculated by site for each fecal source identification genetic marker data set using all samples. To investigate the potential influence of *E*. *coli* and precipitation levels on fecal pollution source trends, water samples from each site were then organized into two groups prior to fecal score determination. For *E*. *coli*, groups included: 1) samples with *E*. *coli* < 410 MPN/100 ml and 2) samples with *E*. *coli* ≥ 410 MPN/100 ml. For precipitation, groups included: 1) 12 h cumulative precipitation < 2.54 mm, and 2) 12 h cumulative precipitation prior to sampling ≥ 2.54 mm.

#### Land use trend analysis

Correlation analyses were performed between land use metrics [impervious surface, park, and residential areas (km^2^)] and site ranking fecal scores (all samples from a site) or fecal scores generated from *E*. *coli* and precipitation sample groupings. Correlation analyses were only determined for combinations yielding eligible fecal scores for all sites. A significant land use trend was defined as any correlation combination yielding an *R*^2^ ≥ 0.6 with p ≤ 0.05. All statistics were conducted with SAS software (Cary, NC) or WinBugs (Version 1.4.3; https://www.mrc-bsu.cam.ac.uk/software/bugs/).

## Results

### Precipitation

**Fig 2:**
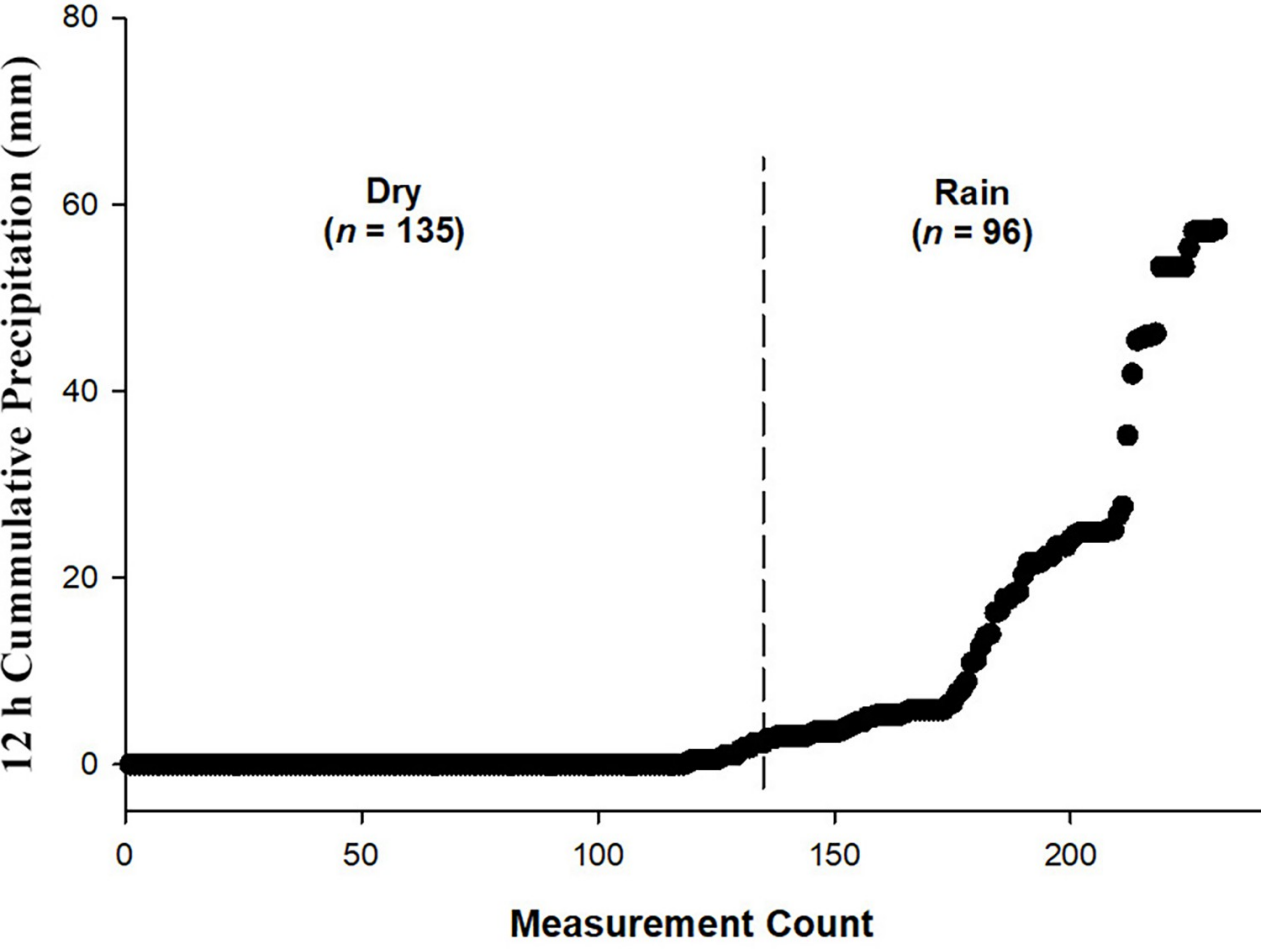
Precipitation measurements (*n* = 231) ranked from lowest to highest quantities (values increase from left to right). The vertical dashed line denotes number of samples with precipitation level < 2.54 mm.

Precipitation was determined for each site and sampling event using a rain gauge in close proximity to the study area (Fig 1). Precipitation measurements ranged from 0 mm to 57.4 mm (Fig 2) with 41.6% of samples designated as rain event samples (*n* = 96; ≥ 2.54 mm).

### Land use metrics

Impervious surface, park, and residential area land use metrics were determined by GIS to investigate potential links to fecal pollution source trends (Table 1). Site catchments ranged in size from 0.13 km^2^ (Alger Park) to 1.23 km^2^ (Fort Dupont). Impervious surface and park areas exhibited a strong positive correlation (*R*^2^ = 0.92). A gradient of impervious surface area was observed spanning 0.04 km^2^ (Alger Park) to 0.22 km^2^ (Fort Chaplin) (Fig 1). In contrast, the largest park area was observed at Fort Dupont (1.23 km^2^) followed by Pope Branch (0.80 km^2^).

**Table 1:** Summary of land use measurements (km^2^) for each catchment.

Catchment	Total Area	Park Area	Impervious Surface Area	Residential Area
Alger Park	0.13	0.03	0.04	0.06
Forth Chaplin	0.61	0.11	0.22	0.26
Fort Davis	0.24	0.12	0.05	0.05
Fort Dupont	1.23	1.13	0.09	0.03
Fort Stanton	0.49	0.23	0.12	0.16
Pope Branch	0.80	0.28	0.20	0.27
Texas Run	0.14	0.08	0.02	0.03

### *E*. *coli* measurements

**Fig 3:**
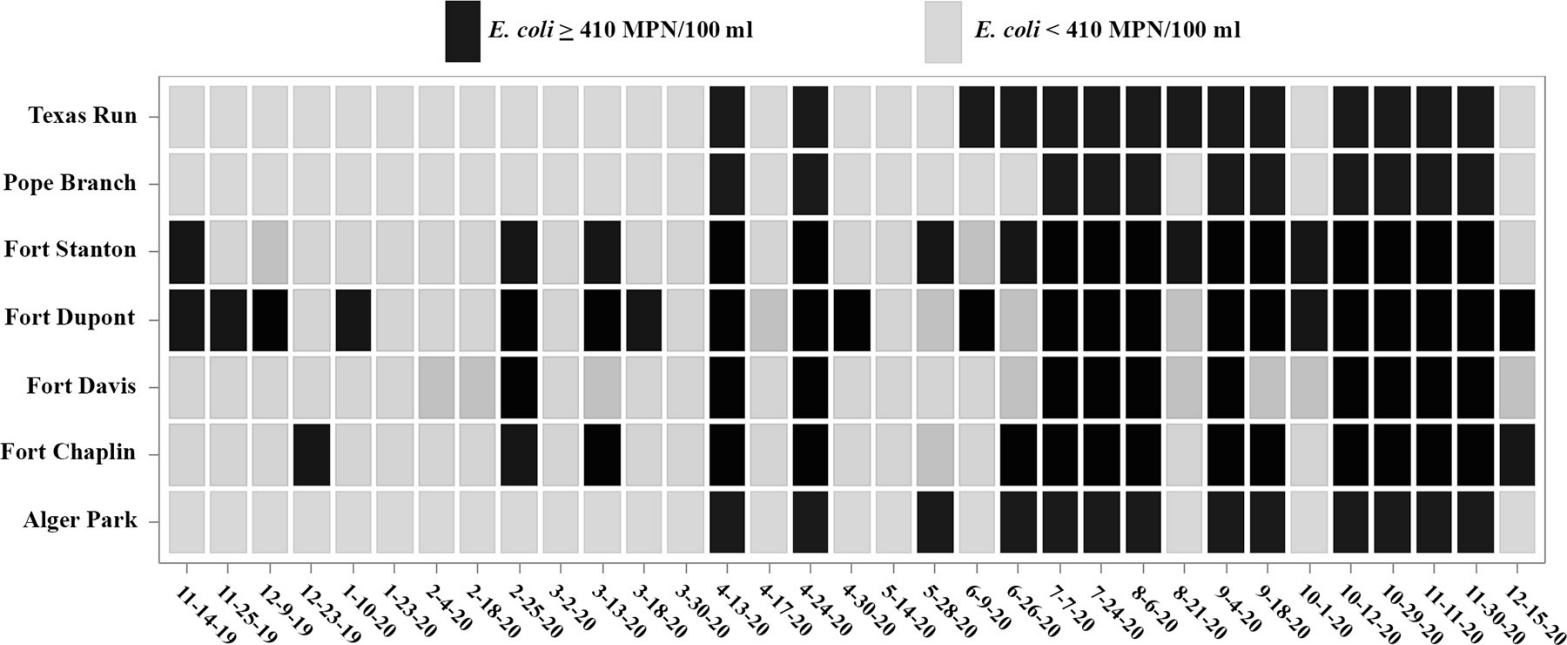
Samples that exceeded the local single sample maximum of *E*. *coli* 410 MPN/100 ml.

*E*. *coli* MPN/100 ml were determined from 231 water samples collected over a 57-week period from seven sampling sites in the Anacostia River Watershed (Fig 1). Log_10_ *E*. *coli* MPN/100 ml summary statistics are shown in Table 2. Measurable levels of *E*. *coli* were present in all samples except one (Fort Chaplin, February 18, 2020) ranging from 0 to 4.74 log_10_ MPN/100 ml across sites. A total of 103 samples (44.6%) exceeded the local single sample maximum value of 410 MPN/100 ml (Fig 3).

**Table 2:** Log_10_ *E*. *coli* (MPN/100 ml) measurements by site.

Site	*n*	Min.	Max.	Median	Mean	SD
Alger Park	33	0	4.08	2.15	3.09	3.39
Fort Chaplin	32	0.93	3.57	2.51	3.65	3.93
Fort Davis	33	1.18	4.26	2.91	3.45	3.67
Fort Dupont	33	1.72	4.46	2.76	3.66	3.85
Fort Stanton	33	0	4.61	2.86	3.54	3.90
Pope Branch	33	0.49	4.04	2.20	3.16	3.41
Texas Run	33	0	4.74	2.00	3.48	3.99

*n* denotes number of samples.

Min. indicates minimum measurement.

Max. represents maximum measurement.

SD signifies standard deviation.

### qPCR quality controls and data acceptance

A series of quality controls were used to identify high-quality fecal source identification qPCR data. Calibration model performance parameters are shown in Table 3. Extraneous DNA control reactions indicated no evidence of contamination (n = 4,275 reactions). Amplification inhibition was not identified in any multiplex IAC HF183/BacR287 or HumM2 experiments. Instrument run-specific IAC proficiency testing yielded a 100% pass rate with NTC VIC C_q_ standard deviations ranging from 0.07 to 0.75 for HF183/BacR287 (acceptance criteria ≤ 1.16) and 0.10 to 0.33 for HumM2 (acceptance criteria ≤ 1.05) [39]. Five water sample DNA extracts failed SPC testing (2.2%; 5 of 231) exhibiting unsuitable matrix interference and were discarded from the study. All instances were from the same site (Texas Run) between February 25 to May 14, 2020. A SPC proficiency test was used to monitor for suitable DNA recovery for each extraction batch. Sketa22 MEB C_q_ standard deviations ranged from 0.03 to 0.58 across 39 batch preparations (acceptance criteria ≤ 0.62) [39].

**Table 3:** Calibration model performance metrics.

Assay	Slope	Intercept Range	LLOQ	*E*
HF183/BacR287	-3.36 ± 0.019	36.9 to 37.5	33.7 to 34.2	0.98
HumM2	-3.33 ± 0.024	39.6 to 40.1	36.4 to 36.9	1.00
Rum2Bac	-3.42 ± 0.031	40.5 to 41.1	37.1 to 37.8	0.96
DG3	-3.40 ± 0.025	36.8 to 37.3	33.4 to 34.0	0.97
GFD	-3.32 ± 0.018	36.3 to 36.6	33.0 to 33.4	1.00

LLOQ represents lower limit of quantification.

*E* indicates amplification efficiency [*E* = 10^(-1/slope)^ -1].

### Host-associated qPCR measurements

**Fig 4:**
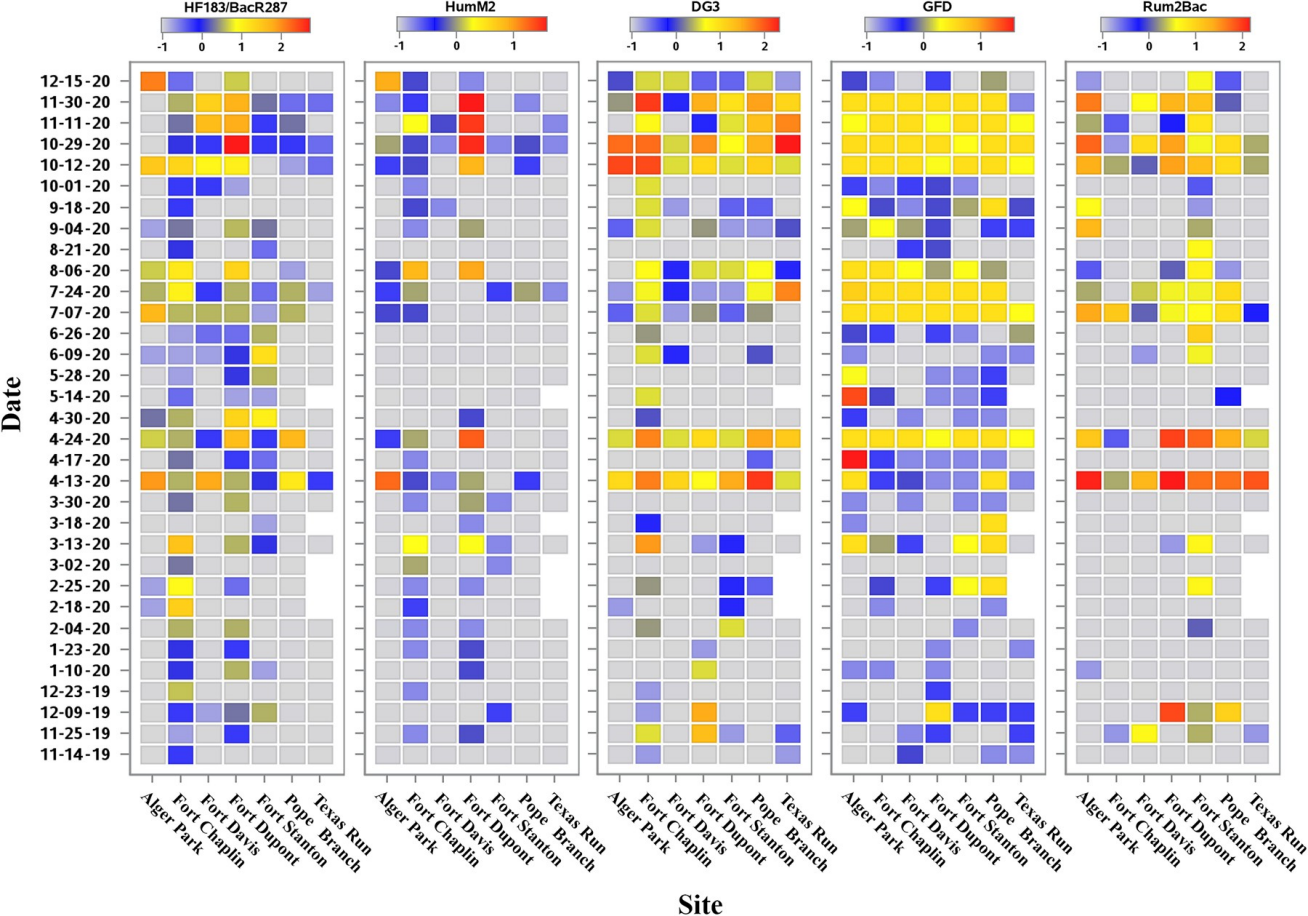
Host-associated qPCR genetic marker estimated log_10_ copies per reaction concentrations for HF183/BacR287, HumM2, DG3, GFD, and Rum2Bac. Heatmap keys are shown for each qPCR assay data set indicating estimated log_10_ copies per reaction color coding information. Sampling date and site combinations denoted by a white cell represents samples that failed the sample processing control and were discarded from the study. Cells shaded grey indicates samples where all sample replicate reactions yielded a C_q_ = 40 (non-detection).

Host-associated genetic markers were quantified for eligible water samples (*n* = 226) using fecal source identification qPCR methods for human (HF183/BacR287 and HumM2), dog (DG3), avian (GFD), and ruminant (Rum2Bac). Estimated mean log_10_ copies per reaction concentrations are shown in Fig 4. The number of samples where all replicate reactions yield a 40 C_q_ (non-detection) ranged from 79 (34.2%, GFD) to 158 (68.4%, HumM2).

### Host-associated genetic marker fecal scores

**Fig 5:**
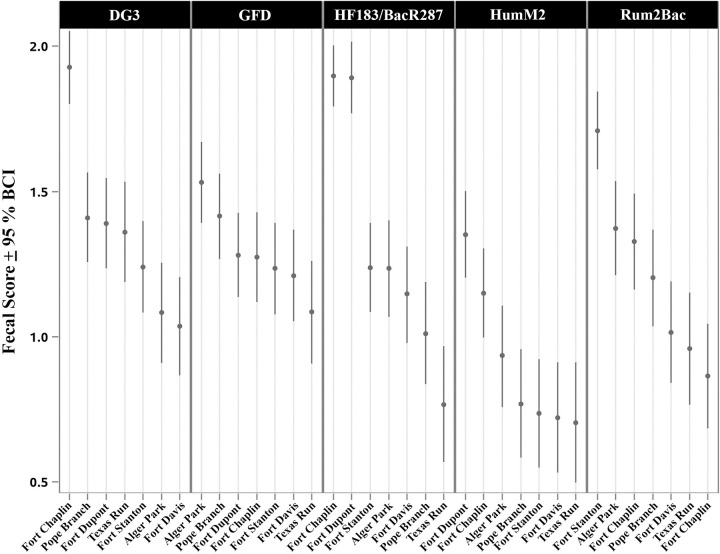
Fecal scores (log_10_ copies per 100 ml with 95% BCI) for site rankings with each host-associated genetic marker. Values are ranked from highest (left) to lowest (right).

For each host-associated genetic marker, site ranking fecal scores (log_10_ copies per 100 ml with 95% BCI) are shown in Fig 5. The Fort Chaplin site ranked in the top three for human, dog, and ruminant fecal sources. In contrast, Texas Run fecal scores were in the bottom two rankings for avian, human, and ruminant waste. The Fort Stanton site had the highest levels of ruminant fecal waste significantly higher than all other sites (95% BCI do not overlap).

**Fig 6:**
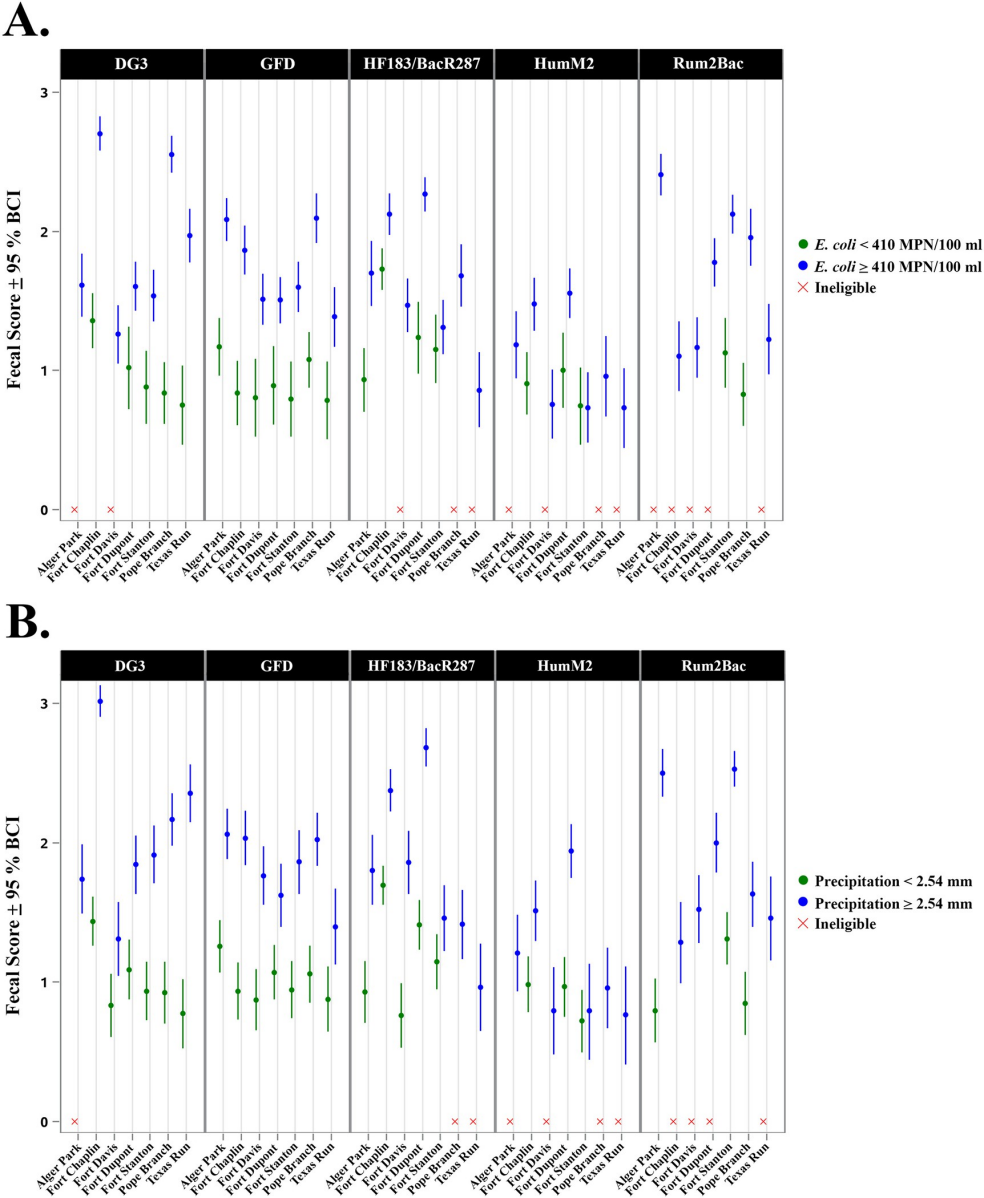
Host-associated genetic marker fecal scores (log_10_ copies per 100 ml with 95% BCI) for each site using *E*. *coli*. (Panel A; threshold: ≥ 410 MPN/100 ml) and precipitation (Panel B; threshold: ≥ 2.54 mm) sample group definitions. An ‘X’ denotes that a sample grouping was ineligible for fecal score determination.

Fecal scores for each site using *E*. *coli* (threshold: ≥ 410 MPN/100 ml) and precipitation (threshold: ≥ 2.54 mm accumulation 12h prior to sampling) sample group definitions are shown in Fig 6. Overall, there was a high level of agreement between *E*. *coli* and precipitation sample group fecal score trends. Avian, dog, and ruminant fecal scores were always higher after rain or when *E*. *coli* levels exceeded the local management benchmark. When *E*. *coli* was low (< 410 MPN/100 ml) or there was no rain, 25 of 140 combinations (17.9%) were not eligible for fecal score determination due to high levels of non-detections in sample groups. This trend was most pronounced with ruminant fecal scores where the Rum2Bac genetic marker was almost always undetectable in 71.4% (9 of 14) of site *E*. *coli* and precipitation sample group combinations. Human fecal scores exhibited the most variable trends. HF183/BacR287 and HumM2 were rarely detected at Pope Branch and Texas Run in the absence of rain and when *E*. *coli* levels were low. In contrast, human-associated fecal scores from Fort Chaplin, Fort Stanton, Fort Dupont, Alger Park, and Fort Davis indicate the presence of measurable levels of human waste regardless of *E*. *coli* or precipitation grouping. Finally, the Fort Stanton site indicated no significant difference in human-associated fecal scores (95% BCI overlap) for either sample group definition.

### Land use trend analyses

Two strategies were used to investigate potential links between host-associated qPCR measurements and catchment land use metrics [park, residential, and impervious surface areas (km^2^)]. First, fecal scores generated from all samples at each site indicated no significant correlation to a land use metric (p ≥ 0.06; *R*^2^ ≤ 0.375). Second, samples from each site were then organized into two groups based on *E*. *coli* (≥ 410 MPN/100 ml) or 12h cumulative precipitation (≥ 2.54 mm) threshold definitions. Only one combination yielded a significant correlation: avian-associated fecal scores (precipitation group = samples ≥ 2.54 mm) and residential area (km^2^) (p = 0.038; *R*^2^ = 0.612). All human-, dog-, and ruminant-associated fecal scores exhibited no significant land use trends (p > 0.05).

## Discussion

Municipal stormwater outfall discharges may contain fecal waste originating from a variety of sources making it challenging to manage, especially when monitoring is limited to general fecal indicators such as *E*. *coli*. This study investigates paired measurements of five host-associated genetic markers indicating human, ruminant, dog, and avian fecal pollution sources with *E*. *coli* and precipitation from seven headwater urban streams that receive discharge from multiple stormwater outfalls. Findings demonstrate the benefits of combining fecal source identification with *E*. *coli* monitoring, provide new insights on host-associated genetic method selection and performance in urban catchments impacted by stormwater outfalls, and further refines a recently reported censored data approach for interpreting qPCR measurements.

### General fecal indicator pollution trends

General fecal indicator bacteria such as *E*. *coli* are routinely used to assess whether fecal pollution is present in urban streams impacted by stormwater discharge. Using a single sample maximum value (410 MPN/100 ml), 44.6% of receiving water samples exceeded the local water quality assessment benchmark demonstrating that these urban streams frequently harbor fecal pollution levels that compromise water quality. In addition, *E*. *coli* levels were 3.57 log_10_ MPN/100 ml or higher across sites after rain events corroborating other research reporting that fecal pollution from urban landscape run-off and stormwater outfalls contribute substantially to local water impairment [41, 42]. These findings provide evidence that there are significant issues with stormwater in the study area but provide minimal information on potential pollutant sources. As a result, it remains unclear whether *E*. *coli* originate from human waste, resident pet owner practices, local wildlife, or some combination of sources demonstrating that paired measurements of host-associated genetic markers could enhance water quality management.

### Human fecal pollution trends

Human fecal pollution is commonly identified in urban stormwater [43–45] and can represent a considerable public health risk when present at unsafe levels [46, 47]. In this study, human waste fecal pollution trends were characterized using recently nationally validated HF183/BacR287 and HumM2 qPCR procedures [34, 35, 39] and SRM 2917 [31, 32], revealing multiple trends. First, human fecal pollution was detected at all sites, but occurrence was highly variable ranging from 9.1% (Texas Run) to 96.7% (Fort Chaplin) of samples suggesting that mitigation strategies will likely vary from one site to another. Second, human fecal pollution average concentrations were significantly higher (fecal score 95% BCI do not overlap) in samples where *E*. *coli* levels exceeded the 410 MPN/100 ml benchmark or after rain events indicating a close link between human waste, precipitation, and reduced water quality, except the Fort Stanton site. At this site, fecal scores were not significantly different (fecal score 95% BCI overlap) regardless of sample groupings even though 42.4% of samples (*n* = 14) indicated no detection of HF183/BacR287 or HumM2 genetic markers. An inspection of this catchment revealed a stormwater outfall discharging gray water indicating that the fecal score trends observed at this site may be indicative of an illicit connection to a stormwater drainage system. Finally, human-associated genetic results exhibited good agreement between HF183/BacR287 and HumM2 paired measurements. Fort Chaplin and Fort Dupont site samples demonstrated the highest average levels of human fecal pollution based on site ranking fecal scores regardless of human-associated genetic marker. Both methods also identified Texas Run to have the lowest human waste concentration on average. Almost identical trends were observed when samples were grouped by *E*. *coli* or rainfall fecal score definitions. Any differences were likely attributed to the well documented lower sensitivity of the HumM2 qPCR assay [48, 49] leading to a higher occurrence of non-detections in this study (68.4% for HumM2 compared to 48.1% for HF183/BacR287). A higher percentage of non-detections reduces fecal score values, and in extreme cases, renders a sample grouping ineligible for fecal score determination. Due to the high level of agreement and reduced HumM2 sensitivity, future studies may consider using only the HF183/BacR287 qPCR assay (EPA Method 1696.1) to reduce expenses.

### Dog fecal pollution trends

There is a growing body of evidence indicating that pet owner waste management, or the lack thereof, can directly impact local water quality [6, 17, 25]. Antibiotic resistant bacteria [50] as well as some human pathogens and parasites are shed in dog fecal matter [51–53] and dog waste has been reported in stormwater discharges [10, 54, 55]. In District of Columbia urban stream samples, dog-associated genetic marker fecal scores were always higher after rain or when *E*. *coli* levels exceeded the local water quality assessment benchmark. In addition, no significant correlations were observed between fecal scores and impervious surface, park, or residential land use metrics (p > 0.05) suggesting that dog waste management practices may help improve water quality regardless of location. Dog fecal pollution was most pronounced in the Fort Chaplin catchment yielding a significantly higher fecal score (95% BCI does not overlap) compared to all other study sites making this a candidate location to implement improved dog waste management practices such as installation and upkeep of dog waste stations combined with a public awareness campaign. It is also interesting to note that the ability of the DG3 assay to delineate between dog (*Canis lupus familiaris*) and coyote (*Canis latrans*) fecal waste remains unknown. As a result, it is possible that coyotes in the District of Columbia could be contributing to these trends. However, coyote populations in this area are reported to be small with sightings extremely rare [56]. Additional research is needed to confirm the specificity of the DG3 assay and characterize the local coyote population in the study area.

### Wildlife fecal pollution trends

Fecal pollution originating from wildlife can be a difficult challenge for water quality and stormwater managers. The District of Columbia is a highly urbanized city consisting of approximately 20% park land with a large amount of river shoreline providing habitats that can sustain bird and deer populations year-round. These animals shed fecal indicators such as *E*. *coli* and may harbor public health relevant pathogens as well as antibiotic resistance bacteria [57–60]. There is limited information on the occurrence of wildlife waste in urban areas, especially for ruminants [54, 55]. Prior to this study, the influence of wildlife on urban receiving water quality in the District of Columbia remained largely unknown. Findings suggest that wildlife fecal waste exhibit clear links to rainfall, land use, and urban stream water quality impairment in the study area. Results indicate that both avian and ruminant sources were always significantly higher (95% BCI do not overlap) after it had recently rained or when *E*. *coli* levels exceeded the local management benchmark. However, ruminant waste was often not detected in the absence of rainfall or when *E*. *coli* levels were low (9 of 14 fecal score combinations, 64.3%). Avian fecal scores exhibited a different trend where waste was detected regardless of sample groupings suggesting that birds have consistent access to urban streams. In addition, avian fecal scores were the only pollutant source yielding a significant correlation to land use indicating that after rain events, levels rise as the amount of residential area increases in a catchment (p = 0.038; *R*^2^ = 0.612) supporting the notion that microorganisms from bird waste can occur in roof-captured rainwater [61] and other residential surfaces. Together, wildlife fecal source identification findings suggest a catchment loading potential, in which waste builds up on surfaces between rain events and is washed off during a subsequent storm contributing to poor water quality in urban streams.

### Implications for urban receiving water and stormwater management

Findings have multiple implications for the management of urban receiving waters impacted by stormwater discharges. First, experiments demonstrate the utility of fecal source identification to help locate *E*. *coli* sources and identify actionable outcomes that can reduce or eliminate specific pollution sources. For example, host-associated genetic marker findings suggest that the elimination of human waste sources alone may not reduce *E*. *coli* to an acceptable level due to the presence of dog, ruminant, and avian sources. However, prioritizing sites with the highest average concentration of human waste may be an effective strategy to minimize potential exposure to human pathogens reducing public health risks. Site prioritization based on non-human sources can also be helpful to identify where the implementation of pet owner and wildlife mitigation programs will make the largest impact. Second, no sample amplification inhibition and minimal DNA recovery bias (2.2%) suggests that qPCR methods can be a reliable tool for urban streams impacted by municipal stormwater discharges, even in systems with very high levels of fecal pollution after large storm events. Results also suggest that it may not be necessary to include both HF183/BacR287 and HumM2 in future studies, reducing the cost of human fecal source identification implementation. Third, human fecal pollution trends were characterized with nationally validated protocols (EPA Methods 1696.1 and 1697.1) in conjunction with a NIST certified reference material (SRM 2917) allowing for a more direct comparison of measurements presented here with future study data sets that use the same standardized methodology. Fourth, fecal source identification testing of chronically polluted urban streams resulted in data sets with non-detections ranging from 34.2% (GFD) to 68.4% (HumM2) preventing the use of traditional statistical analysis methods. The censored data fecal score approach allowed for the quantification of host-associated genetic marker measurements providing useful insights on the link between pollutant sources, precipitation, land use, and water quality, despite high frequencies of non-detections. Finally, the scope of this study was restricted to urban receiving waters, therefore, additional research is needed to characterize fecal sources directly from stormwater outfalls in the study area.
